# Screening a new European hake (*Merluccius merluccius*) chromosome-level genome assembly suggests an XX/XY sex-determining system driven by the SRY-box transcription factor 3 (*sox3*)

**DOI:** 10.1093/g3journal/jkaf127

**Published:** 2025-06-09

**Authors:** Paulino Martínez, Laura Casas, Natalia Petit-Marty, Andrés Blanco, Maialen Carballeda, Nair Vilas-Arrondo, Jessica Gómez-Garrido, Fernando Cruz, Julio Valeiras, Tyler Alioto, Fran Saborido-Rey

**Affiliations:** Department of Zoology, Genetics and Physical Anthropology, Universidade de Santiago de Compostela, 27002 Lugo, Spain; Instituto de Investigaciones Marinas (IIM-CSIC), 36208 Vigo, Spain; Instituto de Investigaciones Marinas (IIM-CSIC), 36208 Vigo, Spain; Department of Zoology, Genetics and Physical Anthropology, Universidade de Santiago de Compostela, 27002 Lugo, Spain; Department of Zoology, Genetics and Physical Anthropology, Universidade de Santiago de Compostela, 27002 Lugo, Spain; Centro Oceanográfico de Vigo, Instituto Español de Oceanografía (IEO-CSIC), 36390 Vigo, Spain; Centre Nacional D'Anàlisi Genòmica (CNAG), 08028 Barcelona, Spain; Universitat de Barcelona, 08028 Barcelona, Spain; Centre Nacional D'Anàlisi Genòmica (CNAG), 08028 Barcelona, Spain; Universitat de Barcelona, 08028 Barcelona, Spain; Centro Oceanográfico de Vigo, Instituto Español de Oceanografía (IEO-CSIC), 36390 Vigo, Spain; Centre Nacional D'Anàlisi Genòmica (CNAG), 08028 Barcelona, Spain; Universitat de Barcelona, 08028 Barcelona, Spain; Instituto de Investigaciones Marinas (IIM-CSIC), 36208 Vigo, Spain

**Keywords:** sex-determination evolution, genetic differentiation, whole-genome sequencing, MassARRAY, sexual conflict, intraspecific variation, genome assembly

## Abstract

Sex determination is exceptionally diverse and shows a high evolutionary rate in fish. European hake (*Merluccius merluccius*) is a species of great commercial value distributed throughout European coasts, which displays a significant sexual growth dimorphism. We present a chromosome-level genome of *M. merluccius*, composed of 215 contigs using long- and short-read sequencing, further scaffolded into the species’ 21 chromosomes using Hi-C technique (715 Mb). RNA-seq on several tissues from pooled individuals improved annotation (26,625 protein-coding genes and 11,083 ncRNAs). Five males and 5 females from an Atlantic population were re-sequenced at 30× depth to look for association with sex across the whole genome. Genetic differentiation between males and females (*F*_ST_) and intrapopulation fixation index (*F*_IS_) pointed to a region on chromosome 9 spanning ∼10 Mb which included several genes related to gonad differentiation and showed strong linkage disequilibrium associated with a putative inversion. Near *sox3* gene (∼25 kb), SNPs were mostly heterozygous in males and homozygous in females, consistent with an XX/XY sex-determining (SD) system. These SNP markers were validated in a larger sample of 56 males and 63 females from the same population using MassARRAY. Other genomic regions that were differentiated between males and females and suggestive of sexual conflict were also explored across the genome. Results support a candidate master SD gene in *M. merluccius* and indicate some differentiated regions potentially under sexual conflict. This information will be useful for the fisheries management of *M. merluccius* in the context of climate change, where noninvasive sex identification tools are essential.

## Introduction

Sex determination (SD) refers to the mechanism controlling the fate of the undifferentiated gonadal primordium during early-life stages, ultimately responsible for the sex of a mature individual. Initially, SD was associated with chromosomal heteromorphisms due to the highly conserved SD systems studied in *Drosophila*, mammals, and birds. However, increasing information from ectothermic vertebrates has revealed a much more diverse scenario (Martínez *et al*. 2014; Guiguen *et al*. 2019). Chromosomal heteromorphisms associated with sex are rare in fish, even within the highly diverse neotropical ichthyofauna (Cioffi *et al*. 2017), and opposite SD systems are not infrequent between congeneric species (Martínez *et al*. 2014). XX/XY systems are almost twice as frequent as ZW/ZZ in fish, but chromosomal heteromorphisms are more common in the latter (Wang *et al*. 2024).

According to a recent review by Kitano *et al*. (2024) , up to 27 different master SD (MSD) genes have been consistently identified to date across 113 fish species spanning 19 orders. Slightly lower figures have been reported by Wang *et al*. (2024) who identified 19 MSD genes among 140 fish species. While some genes, such as *amh* and its receptors, as well as *dmrt*, have been repeatedly and independently recruited throughout fish evolution, several other MSD genes are species or taxa specific, such as *fshr* in *Solea senegalensis*, *hsd17b1* in the genus *Seriola*, or *sdY* in salmonids. Although most MSD genes are associated with transcription factors at the top of the SD cascade or with genes involved in the TGF-β signaling pathway, recent data suggest that steroidogenesis, a crucial pathway in gonad and sex differentiation, has also been recurrently co-opted for MSD recruitment (Kitano *et al*. 2024). The growing body of knowledge on SD in fish has been facilitated by the development of highly contiguous and reliable chromosome-level genome assemblies used as references for high-throughput SNP genotyping screening facilitated by advances in sequencing technologies and new scaffolding and bioinformatic approaches (Ramos and Antunes 2022).

To understand the evolutionary dynamics of SD in fish, intraspecific variation should be explored to identify transitions between SD systems (Bachtrog *et al*. 2014; Martínez *et al*. 2014). For this approach, the genetic structure and degree of isolation of populations are essential reference points. According to theory, sexual conflict at specific genomic regions could trigger a shift to a new SD system to restore population fitness (Van Doorn and Kirkpatrick 2007). Ongoing sexual conflicts associated with transitions between SD systems have been shown in cichlids (Roberts *et al*. 2009), and intrapopulation variation in SD has been reported in fish species such as Northern pike (*Esox lucius*; Pan *et al*. 2021) and gray mullet (*Mugil cephalus*; Ferraresso *et al*. 2021). Different mechanisms have been shown to underlie the emergence of new SD genes in fish, including gene duplication or the presence of alternative alleles at the MSD gene (Kitano *et al*. 2024). To clearly elucidate SD mechanisms and regions under sexual conflict, comprehensive chromosome-level assemblies and genome-wide genotyping data are required (de la Herrán *et al*. 2023).

The European hake (*Merluccius merluccius*) is widely distributed in the Mediterranean Sea and the Northeast Atlantic Ocean, ranging from the coasts of Norway and Iceland to the Guinea Gulf (Murua 2010). Its range also extends eastward into the North Sea, Skagerrak and Kattegat, and occasionally into the Black Sea (Casey and Pereiro 1995; Kutsyn *et al*. 2024). It is considered one of the most important commercial species in both the Northeast Atlantic and the Mediterranean, where populations have been extensively and intensively harvested, leading to its classification as overexploited (FAO, 2020; Izquierdo *et al*. 2021; Morales-Nin *et al*. 2022). The European hake is a gonochoristic species that exhibits significant sexual size dimorphism, with females reaching greater sizes and weights than males. Thus, from 40 to 60 cm, the proportion of males decreases progressively, and above 70 cm, females account for 100% of the individuals in the Atlantic Iberian waters, where they have historically reached lengths of up to 130 cm (Piñeiro and Saínza 2003; Domínguez-Petit and Saborido-Rey 2010; Cerviño 2014; El Bouzidi *et al*. 2022; Apostologamvrou *et al*. 2023). This is the consequence of the differential maturation pattern between sexes influencing growth and natural mortality, which in turn affects population dynamics and stock productivity (Cerviño 2014). Consequently, having a cost-effective tool available to sex hake specimens for stock monitoring would be important for fisheries management.

The available genome assembly of *M. merluccius* when our study started was highly fragmented (N50: 5.1 kb), with a coverage below its expected size (401 Mb), and additionally, lacked functional annotation (PRJEB12469; March 2018; scaffold N50: 5.1 kb; total length: 401 Mb). Consequently, a first goal of our study was to generate not only a high-quality and contiguous chromosome-level genome assembly of *M. merluccius* to investigate its SD system but also an essential genomic resource for fisheries management for this important commercial species. Several males and females from an Atlantic population were screened across the whole genome using millions of SNPs to identify regions associated with sex. Detailed analysis of the most significant regions was conducted to identify candidate MSD genes, and the findings were validated in a larger sample of sexed specimens. Furthermore, other genomic regions that differentiated between males and females were inspected and validated as potential sexual conflict regions. Results suggest an SD XX/XY system in European hake driven by the SRY-box transcription factor 3 (*sox3*).

## Materials and methods

### Sampling

A single *M. merluccius* female from the Spanish Atlantic Ocean (Galician shelf) was used for genome assembly. Additionally, pooled samples from 5 different tissues (brain, muscle, gonad, spleen, and liver) from 3 individuals of the same population were used for RNA-seq to improve genome annotation. Five males and 5 females from the same Atlantic population were collected to explore sex association using whole-genome sequencing (WGS). To validate the candidate SD region, 56 males and 63 females from the same Atlantic population were analyzed using MassARRAY genotyping on a selected SNP subset. Biological and genomic metadata for the *M. merluccius* samples analyzed in this study are provided in Supplementary Table Metadata.

### DNA and RNA sequencing

#### Long-read WGS

High molecular weight DNA was purified from the spleen of a single female of *M. merluccius* using the Nanobind CBB Big DNA Kit (Circulomics), following the manufacturer's instructions, and eluted in EB buffer (Qiagen). Sequencing libraries were prepared with the ligation sequencing kit SQK-LSK110 from Oxford Nanopore Technologies (ONT). Sequencing runs were carried out on a PromethION 24 using a FLO-PRO002 Flowcell. The quality parameters of the sequencing runs were monitored in real time by the MinKNOW core version 5.1.0, and base calling was performed with Guppy version 6.1.5.

#### Short-read WGS

The short-insert paired-end libraries for WGS were prepared from the DNA of the same female used for long-read sequencing, following a PCR-free protocol with some modifications using the KAPA HyperPrep kit (Roche).

#### RNA-seq

Total RNA extraction was performed with the RNeasy mini kit (Qiagen) with DNase treatment. RNA quantity and quality were evaluated with the Qubit RNA BR Assay kit (Thermo Fisher Scientific), and RNA integrity was estimated using the RNA 6000 Nano Bioanalyser 2100 Assay (Agilent; RNA integrity number >9). Next, equimolar RNA pools from 3 individuals were used for library construction for each tissue (brain, liver, muscle, spleen, and gonad) following quality evaluation of individual RNA extractions. RNA-seq libraries were prepared with the KAPA Stranded mRNA-Seq Illumina Platforms Kit (Roche) following the manufacturer's recommendations.

The short-read WGS and RNA-seq libraries were sequenced on a NovaSeq 6000 (Illumina) in paired-end mode, with a read length of 151 bp for WGS and 100 bp for RNA-seq, following the manufacturer's protocol for dual indexing. Image analysis, base calling, and quality scoring of the run were processed using the manufacturer's software Real Time Analysis (RTA 3.4.4), followed by the generation of FASTQ sequence files.

#### Hi-C sequencing

Two females and 1 immature specimen were dissected, and 5 different organs (brain, muscle, liver, spleen, and gonads) extracted and stored at −80°C. Organs were pooled and pulverized using a mortar and pestle immersed in a liquid nitrogen bath before Hi-C library preparation following the Omni-C kit (Dovetail Genomics) protocol. The library was sequenced on a NovaSeq 6000 (Illumina, 2 × 151 bp) following the manufacturer's protocol for dual indexing.

### Genome assembly

The genome was assembled using long-read WGS from ONT, Illumina paired-end reads to improve base accuracy, and Omni-C contact data to map sequences to chromosomes using the CLAWS1 pipeline (Supplementary Fig. 1; Gomez-Garrido *et al*. 2024).

Prior to assembly, adaptors present in the Illumina data were trimmed with TrimGalore (https://github.com/FelixKrueger/TrimGalore). A *k*-mer database (*k* = 20) was subsequently built with Meryl (https://github.com/marbl/meryl) using the trimmed short-read data. The *k*-mer histogram generated by Meryl was used as input to GenomeScope2.0 (Ranallo-Benavidez *et al*. 2020) to visualize the *k*-mer distribution and to estimate the haploid genome size, heterozygosity, and repeat content. Adaptor sequence present in the nanopore reads were automatically removed by the base-calling software prior to FASTQ generation. The ONT data were then filtered with Filtlong (https://github.com/rrwick/Filtlong; −minlen 1000 –min_mean_q 80) prior to assembly to remove short and low-quality reads.

**Fig. 1.**
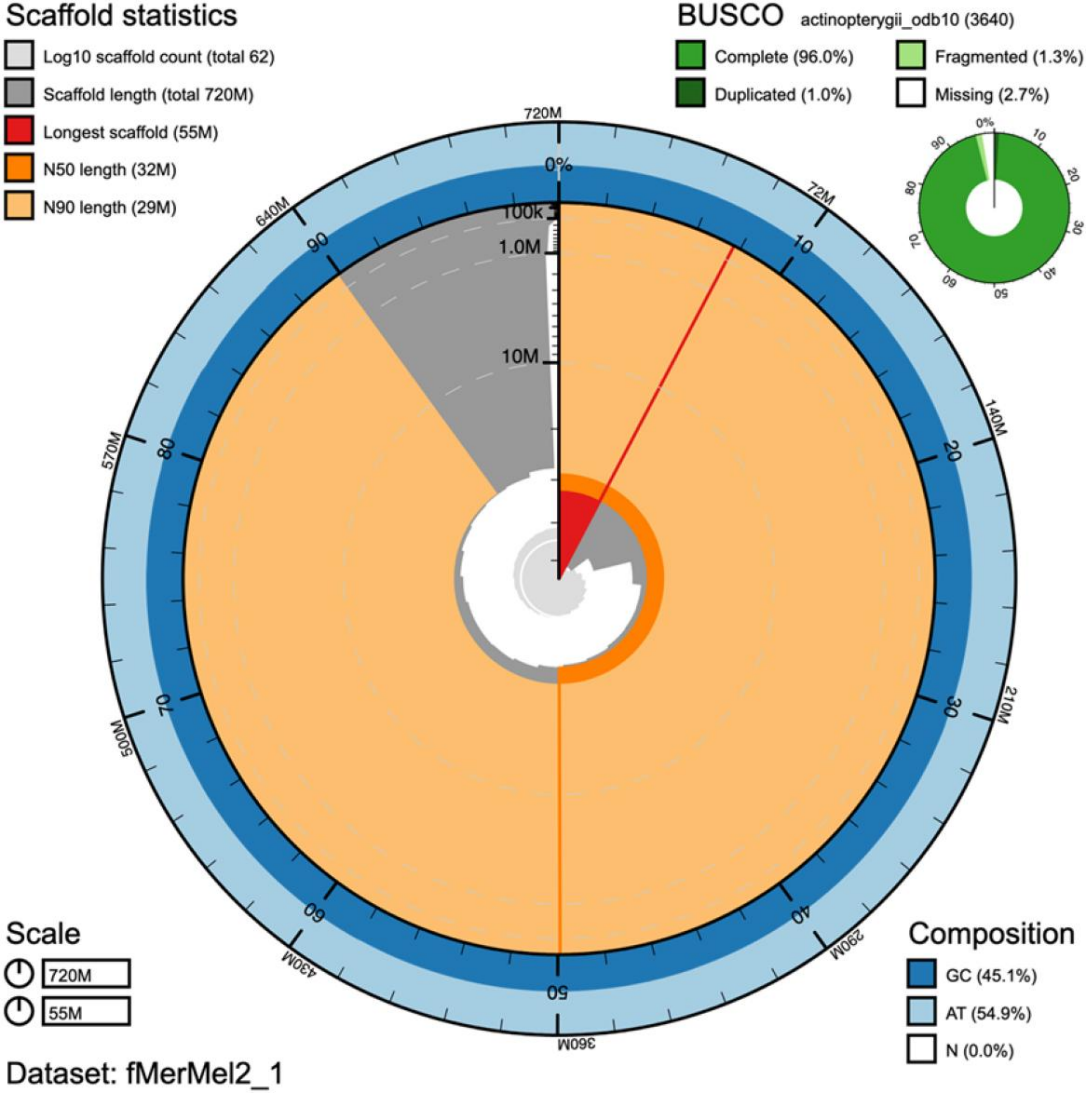
Snail plot showing the main features of the *M. merluccius* genome assembly.

The filtered ONT data were assembled with Nextdenovo v2.5.04 (https://github.com/Nextomics/NextDenovo) using the “nano-raw” mode and a minimum overlap of 1,000 bp. To improve base accuracy, the assembly was polished with HyPo5 (https://www.rcac.purdue.edu/software/hypo), using both Illumina and ONT data. The polished assembly was then purged with purge_dups (Guan *et al*. 2020) to remove alternate haplotypes and other artificially duplicated repetitive regions. The resulting assembly was scaffolded using the Omni-C data with YAHS (Zhou *et al*. 2023). Manual curation of the assembly was performed with PretextView (https://github.com/wtsi-hpag/PretextView). Finally, the Blobtoolkit (Challis *et al*. 2020) pipeline was applied to the scaffolded assembly (Fig. 1), using the NCBI nt database (updated on November 2022) and several BUSCO odb10 databases (Manni *et al*. 2021), including actinopterygii, vertebrata, metazoa, eukaryota, fungi, and bacteria.

### Genome annotation

Repeats in the genome assembly were annotated with RepeatMasker v4-1-2 (http://www.repeatmasker.org) using the custom repeat library available for *Danio rerio*. Additionally, a specific repeat library for our assembly was generated with RepeatModeler v1.0.11. Repeats associated with repetitive protein families were excluded by performing a BLAST1 (Altschul *et al*. 1990) search against the UniProt database. RepeatMasker was then rerun with this new library to annotate the specific repeats.

Gene annotation of the *M. merluccius* genome assembly was achieved by integrating transcript alignments, protein alignments, and ab initio gene predictions using the ANN1 flowchart (Supplementary Fig. 2). RNA-seq data from 5 organs (brain, liver, muscle, spleen, and gonad) were used for annotation. Reads were aligned to the genome using STAR v-2.7.2a (Dobin *et al*. 2013), and transcript models were subsequently generated using Stringtie v2.2.1 (Pertea *et al*. 2015) and merged using TACO v0.7.3 (Niknafs *et al*. 2017). High-quality junctions for annotation were obtained by running ESPRESSO v1.3.0 (Gao *et al*. 2023) after mapping with STAR. Finally, PASA assemblies were produced with PASA v2.5.2 (Haas *et al*. 2008), and TransDecoder program, which is part of the PASA package, was used to identify coding regions in the transcripts. Next, the complete proteomes of *D. rerio*, *Chanos chanos*, and *Carassius auratus* were downloaded from UniProt (May 2023) and aligned to the genome using Miniprot v0.6 (Li 2023). Ab initio gene predictions were performed on the repeat-masked assembly with 3 programs: GeneID v1.4 (Alioto *et al*. 2018), Augustus v3.5.0 (Stanke *et al*. 2006), and Genemark-ET v4.71 (Lomsadze *et al*. 2014), with and without RNA-seq data. Gene predictors were run with human-trained parameters, except for Genemark, which runs in a self-trained mode. Finally, all data were combined into consensus CDS models using EvidenceModeler-1.1.1 (EVM; Haas *et al*. 2008). Additionally, UTRs were identified and alternative splicing forms annotated through 2 rounds of PASA annotation updates. Functional annotation of the annotated proteins was performed with Pannzer's 11 online server (Toronen and Holm 2022).

The annotation of noncoding RNAs (ncRNAs) was obtained by running the following steps on the repeat-masked version of the genome assembly. First, the program cmsearch v1.1 (Cui *et al*. 2016), which is part of the Infernal (Nawrocki and Eddy 2013) package was run against the RFAM database of RNA families v12.0. Additionally, tRNAscan-SE v2.08 (Chan and Lowe 2019) was used to detect transfer RNA genes present in the genome assembly. Long noncoding RNAs (lncRNAs) were identified by first filtering the set of PASA assemblies that were not included in the protein-coding gene annotation, retaining only those transcripts longer than 200 bp and not covered by >80% by a small ncRNA. The resulting transcripts were clustered into genes using shared splice sites or significant sequence overlap as criteria for designation as the same gene.

### Comparison of *M. merluccius* genome assemblies

*Merluccius merluccius* assembly from this study (female; origin: Galicia, NW, Spain) was aligned using minimap2 (Li, 2018) against the chromosome-level assembly recently released (PRJEB77069; September 2024; male; origin: North Sea, Oslofjord, Norway). The resulting PAF format files (Pairwise mApping Format) were plotted by ggplot2 (Wickham 2016) and pafr (Winter 2020) R packages.

### MSD gene candidates

DNA was extracted from fin clip tissue of 5 adult males and 5 adult females originating from an Atlantic population; sequencing was performed using 150 bp PE reads on an Illumina NovaSeq 6000 at 30× depth in the Centre Nacional d'Anàlisi Genòmica (CNAG, Barcelona, Spain) Platform, following the outlined short-read WGS protocol. The reads were filtered using fastp v.0.19.7 (Chen *et al*. 2018), trimming bases with Phred quality <15 and reads with length <30 bp. Each sample was then aligned independently against the newly assembled *M. merluccius* reference genome using Burrows–Wheeler Aligner v.0.7.17 (Li and Durbin 2009), with default parameters.

A large SNP dataset was called and genotyped for 5 males and 5 females using SAMtools v.1.10 (Li *et al*. 2009), and SNPs showing quality scores below 20 were removed. Sampling of a few numbers of males and females for high-throughput screening has been successfully implemented in other species to detect sex-associated genomic regions as a preliminary approach (Martínez *et al*. 2021; De la Herrán *et al*. 2023). This SNP dataset was used to estimate the relative component of genetic differentiation between males and females (*F*_ST_) and the intrapopulation (sex) fixation index (*F*_IS_) across the whole genome using GENEPOP 4.7.5 (Raymond and Rousset 1995). Then, we not only explored the variation of both parameters across each chromosome at single SNP level but also averaged their values across 5, 10, and 20 SNP sliding windows (1 SNP shift between consecutive windows), considering the variation of SNP frequencies. After comparing these strategies, we decided to average *F*_ST_ and *F*_IS_ values over 10 consecutive SNPs to look for deviation from the null hypothesis (*F*_ST_ and *F*_IS_ = 0). Expected *F*_ST_ and *F*_IS_ values for regions consistent with an XX/XY or a ZZ/ZW SD system would be 0.5 and −1, respectively, when partitioning total genetic diversity within and between sexes (Wright 1949). Given that we averaged these values over 10 SNPs for the sliding windows, we established more relaxed thresholds to identify candidate regions (*F*_ST_ ≥ 0.3 and *F*_IS_ ≤ −0.5), considering that not all SNPs within a window would show the same allele frequencies and, particularly, the expected sex-association pattern. Additionally, we only considered genomic windows >1 kb to exclude putative false positives, although these regions were also inspected. Our strategy, successfully implemented in other species (Martínez *et al*. 2021; De la Herrán *et al*. 2023), involves a double check to identify the SD region, based not only on genetic differentiation between male and female populations (as in the pooled DNA strategy) but also on the expected heterozygote excess in the region associated with the heterogametic sex. Finally, we also inspected linkage disequilibrium across the region most associated with sex by estimating *r*^2^ with PLINK v2.0 (Chang *et al*. 2015) and the *P*-value estimated through exact tests for genotyping disequilibrium implemented in GENEPOP 4.7.5.

### Validation of the SD region

To validate the most promising candidate sex-associated genomic regions, we expanded our sample to 56 males and 63 females from the same Atlantic population. DNA was extracted from fin clips stored in pure ethanol using the DNeasy Blood and Tissue Kit (Qiagen) following the manufacturer's instructions. A MassARRAY assay was used for SNP genotyping, utilizing a set of markers located in the candidate regions (selecting at least 2 markers per region showing the closest genotyping pattern consistent with the disclosed XX/XY SD system; see Results). SNP selection for each region was based on technical feasibility and the absence of other polymorphisms within ±100 bp of the SNP, as indicated by the WGS data from the initial 5 males and 5 females. Primer design and genotyping were done at the UCIM-University of Valencia Genomics Platform, using MALDI-TOF mass spectrometry analysis on an Autoflex spectrometer.

## Results

### Genome assembly

The initial assembly obtained with Nextdenovo spanned 724 Mb (245 scaffolds). This assembly was scaffolded using the Omni-C data (Supplementary Fig. 3), and after manual curation, a total of 62 scaffolds were obtained that were assembled at the chromosome-level assembly (716 Mb; 21 chromosomes; Table 1; Supplementary Table 1). The high resolution of Onmi-C scaffolding can be observed in the accurate picture of the 2 arms of several metacentric/submetacentric chromosomes (C) 1, C2, and C14 likely corresponding to the C1, C2, and C15 of the *M*. *merluccius* karyotype (García-Souto *et al*. 2015). The contig and scaffold N50 of the final assembly were 10.9 and 32 Mb, respectively, and 50% of the sequence (L50) comprehended the 10 biggest superscaffolds (Fig. 1; Table 1). The consensus quality (QV) of the final assembly was estimated by Merqury as 42, and the gene completeness reported by BUSCO v5 was 96% using the Actinopterygii_odb10 database.

**Table 1.** Statistics of the genome assembly of *M. merluccius*.

Assembly	Nextdenovo	Nextdenovo + hypo	Nextdenovo + hypo + purged	fMerMel2.1
Contig N50	10,991,627 bp	10,936,441 bp	10,936,441 bp	10,936,441 bp
Scaffold N50	10,991,627 bp	10,936,441 bp	10,936,441 bp	32,021,586 bp
Scaffold L50	21	21	21	10
Total sequences	245	245	215	62
Total length	723,776,638 bp	720,771,172 bp	715,974,550 bp	715,430,492 bp
BUSCO*^a^*	95.2%	96.1%	96.0%	96.0%
BUSCO*^b^*	1.2%	1.3%	1.1%	1%
QV	35	41	42	42
*k*-mer completeness	89.38	90.33	90.27	90.25

BUSCO v5 actinopterygii_odb10 database: *^a^*complete; *^b^*duplicated.

### Genome annotation

Repeated elements represented 43.3% of the *M. merluccius* genome, with DNA elements, LINEs, LTRs, and simple repeats being the most frequent. Also, 9.1% of repeated elements was unclassified (Table 2).

**Table 2.** Statistics of repetitive elements of the *M. merluccius* genome.

Type	Length (bp)	%
LINEs	28,949,807	4.05
SINEs	3,319,899	0.46
LTR elements	29,597,425	4.14
DNA elements	134,914,544	18.86
Satellites	2,913,070	0.41
Simple repeats	30,402,074	4.25
Low complexity	4,429,408	0.62
Unclassified	64,963,785	9.08
Other	10,287,643	1.44
Total	309,777,655	43.30

In total, we annotated 26,625 protein-coding genes that produced 41,543 transcripts (1.56 transcripts per gene) and encoded 37,855 unique protein products (Table 3; Supplementary Table 2a and b). We were able to assign functional labels to 78% of the annotated proteins (Supplementary Table 3). The annotated transcripts contain 11.84 exons on average, with 97% of them being multi-exonic. Additionally, 11,083 noncoding transcripts were annotated, of which 5,683 as lncRNA.

**Table 3.** Statistics of *M. merluccius* genome annotation.

Number of protein-coding genes	26,625
Median gene length (bp)	8,357
Number of transcripts	41,543
Number of exons	285,286
Number of coding exons	266,171
Median UTR length (bp)	1,254
Median intron length (bp)	501
Exons/transcript	11.84
Transcripts/gene	1.56
Multi-exonic transcripts	0.97
Gene density (gene/Mb)	37.21

### Comparison of *M. merluccius* genome assemblies

The chromosome-level genome assembly of *M. merluccius* from this study (female; origin: Galicia, NW Spain; hereafter referred to as MmG) was aligned against the chromosome-level assembly recently released (male; origin: North Sea, Oslofjord, Norway; hereafter referred to as MmN; Supplementary Fig. 4). The higher genome size of MmG compared to MmN (715.4 vs. 647.0 Mb; 10.6% difference) was observed in all chromosomes, mainly at their ends, suggesting a more complete and higher quality assembly. Chromosome numbers were not fully concordant between assemblies, reflecting unequal sequence completeness across chromosomes, ordered by size in each assembly. Interestingly, several inversions were suggested when comparing both assemblies, both not only at the ends (i.e. C2, C4, C7, and C14) but also at the interstitial (C1, C4, C9, and C16) regions of chromosomes (Supplementary Fig. 4; reference MmG). The most notable finding was a large inversion spanning around 20 Mb at C9. While these inversions could result from assembly errors, they might also represent genuine chromosomal polymorphisms in *M. merluccius*.

### Identification of the SD region

We screened the whole genome of 5 males and 5 females of *M. merluccius* using 1,330,537 SNPs [2.2 SNPs/kb; range: 1.9 (C9)–2.5 (C17)] filtered by MAF ≥ 0.2, assuming an underlying XX/XY or ZZ/ZW SD system (expected frequencies of 0.75 and 0.25 for the X/Z and Y/W alleles, respectively; Supplementary Table 4). The joint inspection of average *F*_ST_ and *F*_IS_ using genomic windows of 5, 10, and 20 SNPs across each chromosome rendered quite similar results, so we decided to use 10 SNP windows as in previous studies (Martínez *et al*. 2021; De la Herrán *et al*. 2023). Our strategy enabled the identification of candidate regions associated with sex, where *F*_ST_ and *F*_IS_ were close to the expected values of 0.5 and −1, respectively, under an XX/XY or a ZW/ZZ SD system (Supplementary Table 5; Supplementary Fig. 5). Interestingly, a side effect of this strategy was the potential recognition of centromere locations of several chromosomes, characterized by very negative and consistent *F*_IS_ across a broad region, probably related to the presence of tandem repetitive elements. This pattern is particularly evident not only in 3 metacentric/submetacentric chromosomes (C1, C2, and C16), but also at any of both ends of many acrocentric chromosomes.

**Fig. 2.**
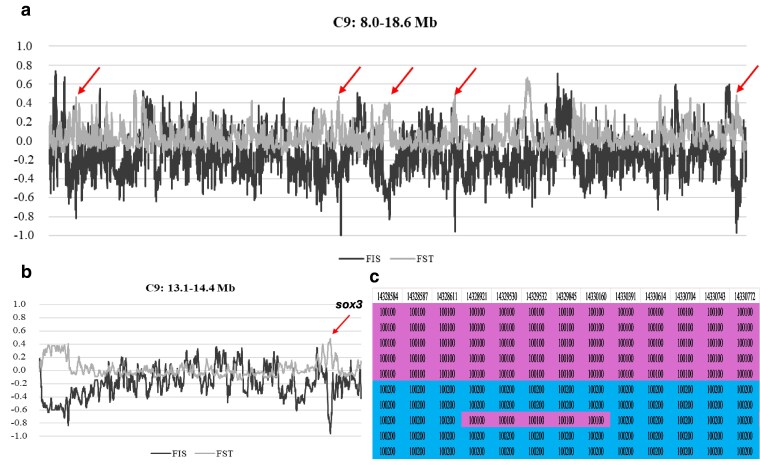
Screening of genetic differentiation (*F*_ST_) and intrapopulation fixation index (*F*_IS_) across chromosome 9 in an Atlantic population of *M. merluccius* subdivided by sex: a) most associated chromosome 9 region; b) zoom where the main MSD gene is located; c) genotypes of 5 female (upper rows; 100,100) and 5 male (bottom rows; mostly 100,120) in the most associated region; arrows point to extreme *F*_ST_ and *F*_IS_ values.

Several regions with *F*_ST_ and *F*_IS_ above or below the cutoff values (0.3 and −0.5, respectively; see Materials and methods) were identified at C13 and C15, but particularly at C9 (Fig. 2a; Table 4). On this chromosome, multiple peaks or subregions with *F*_ST_ and *F*_IS_ values close to those expected under an XX/XY SD chromosome system were detected within a broad region between 8.0 and 18.6 Mb (Fig. 2b). Specifically, a peak of 3.5 kb between 14,323,162 and 14,331,151 bp displayed a high proportion of SNPs that were heterozygous in males and homozygous in females (*F*_ST_ = 0.427; *F*_IS_ = −0.818; Fig. 2c). Similar patterns were observed in other 4 conspicuous subregions on this chromosome with high differentiation between sexes and an excess of heterozygotes in males and homozygotes in females (Supplementary Table 6): (1) 8,443,795–8,451,341 bp (*F*_ST_ = 0.406, *F*_IS_ = −0.748); (2) 12,186,593–12,190,187 (*F*_ST_ = 0.366, *F*_IS_ = −0.500); (3) 18,505,237–18,511,916 bp (*F*_ST_ = 0.384, *F*_IS_ = −0.769); and (4) the broad region of >100 kb between 13,144,747 and 13,249,800 bp (*F*_ST_ = 0.321, *F*_IS_ = −0.572). This consistent pattern suggests the presence of linkage disequilibrium across a substantial region of C9.

**Table 4.** Genomic windows showing *F*_IS_ and *F*_ST_ values above the threshold established to be putatively associated with an SD region.

Chromosome	Position		Window size	*F*_IS_	*F*_ST_
	Start	End			
5	23,555,964	23,563,912	7,948	−0.159	0.535
9	**8,443,795**	**8,451,341**	**7,546**	**−0**.**748**	**0**.**406**
	**12,186,593**	**12,190,187**	**3,594**	**−0**.**500**	**0**.**366**
	**13,144,747**	**13,249,800**	**105,053**	**−0**.**572**	**0**.**321**
	**14,323,162**	**14,331,151**	**7,989**	**−0**.**756**	**0**.**383**
	**18,505,237**	**18,511,916**	**6,679**	**−0**.**769**	**0**.**384**
11	15,533,034	15,534,670	1,636	−0.441	0.369
12	18,533,683	18,536,488	2,805	−0.268	0.489
	24,646,173	24,650,336	4,163	−0.165	0.586
13	13,184,786	13,185,908	1,122	−0.446	0.473
	**14,750,250**	**14,752,940**	**2,690**	**−0**.**550**	**0**.**482**
15	**12,366,138**	**12,371,351**	**5,213**	**−0**.**864**	**0**.**429**
	16,168,025	16,169,465	1,440	−0.44	0.414
19	17,077,934	17,079,795	1,861	−0.466	0.448

Regions meeting both criteria are highlighted in boldface.

Several genes associated with SD or gonadal differentiation were identified within or very close to some of those regions at C9: (1) androgen receptor alpha (*ar*) and progesterone receptor-like (*pgr*); (2) glucocorticoid receptor (*nr3c1*); and (3) motile sperm domain containing 1 (*mospd*), forkhead box O4 (*fox4*), testis expressed 11 (*tex11*) (Supplementary Table 7). However, the most compelling candidate gene associated with the highest differentiated region was the SRY-box-containing gene 3 (*sox3*: 14,303,025–14,303,945 bp). This gene mapped at <25 kb of the most associated region at C9 consistent with an XX/XY system (14,327,382–14,330,907 bp; Supplementary Table 7). To explore a possible gene duplication of *sox3*, we compared the number of reads of this gene in the 5 males and the 5 females. No significant differences were detected, ruling out a male-specific duplication of *sox3*.

### Potential sexual conflict regions

We also explored other regions showing high genetic differentiation between males and females (*F*_ST_ > 0.5) across the *M. merluccius* genome, irrespective of their *F*_IS_ values, using the same sample of males and females (Table 4). Our intention was to identify and validate differentiated genomic regions with allelic variants potentially advantageous to either males or females, reflecting a putative sexual conflict. We initially preselected a list of such 14 regions and mined the genome ±100 kb around them looking for clusters of genes with functions potentially specialized by sex.

We finally retained one region at C5, including a large cluster of genes related to olfaction and chemoreception, and another at C12 mostly related to immunity, to be validated in a broader sample (Table 4; Supplementary Tables 5 and 7; Supplementary Fig. 5). Among the genes in the C5 olfactory cluster (23.522 and 23.815 Mb), we identified several isoforms of phosphoinositide phospholipase C, olfactory receptor C family, v3, olfactory receptor C family, r1, olfactory receptor family C subfamily 11 member 4, olfactory receptor C family, w1 and 1 pheromone receptor. Among the genes in the C12 cluster, we identified E3 ubiquitin-protein ligase, HECT domain containing 1, HEAT repeat containing 5a, heat shock protein nuclear import factor hikeshi, dehydrogenase/reductase SDR family member 13-like and several isoforms of 4F2 cell-surface antigen heavy chain like.

### Validation of the SD region and potentially sexual conflict regions

**Fig. 3.**
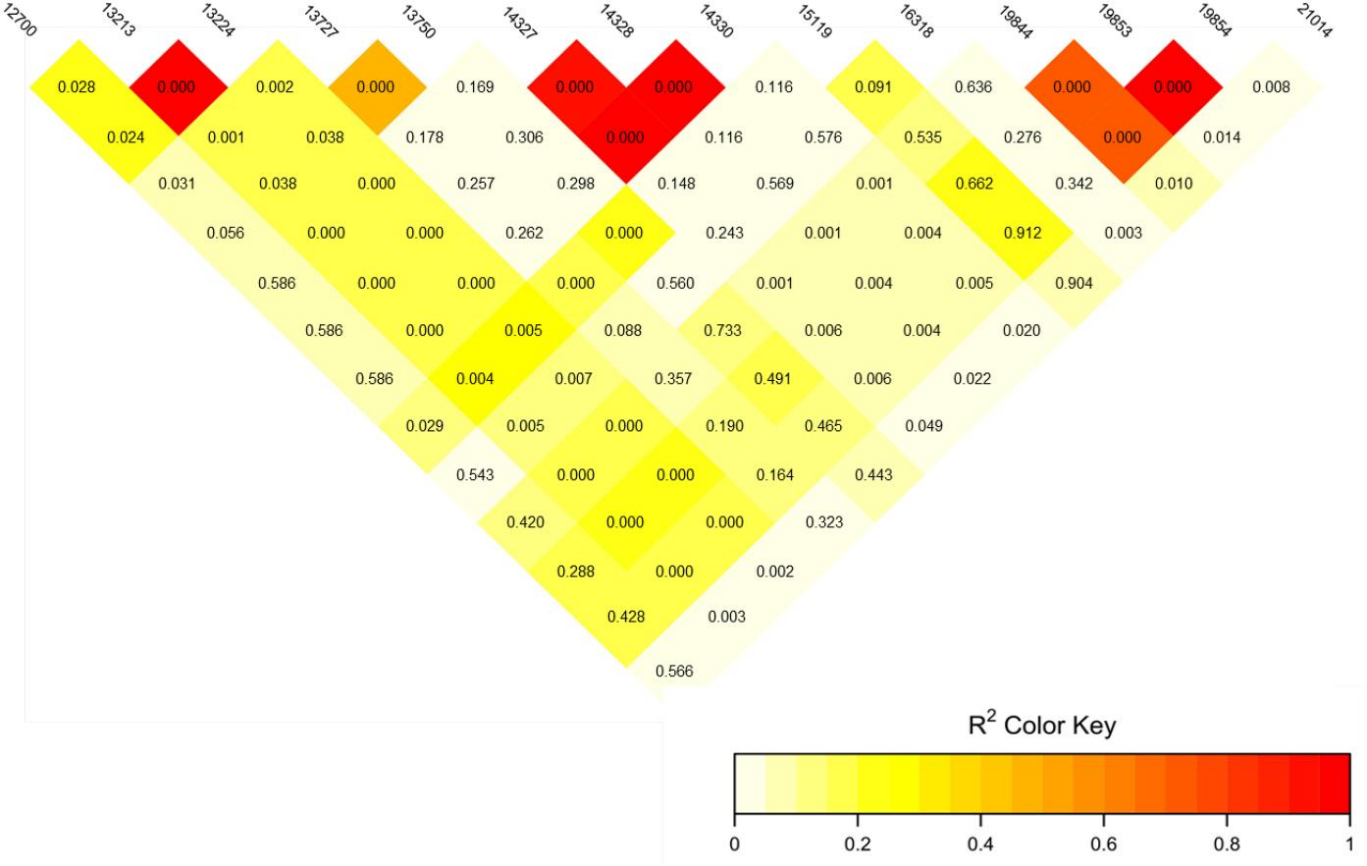
Linkage disequilibrium *r*^2^ values (color scale) and their *P*-values between pairs of loci across the most consistent region associated with sex at chromosome 9 of *M. merluccius*. The codes of SNPs represent their position in Mb across C9.

**Fig. 4.**
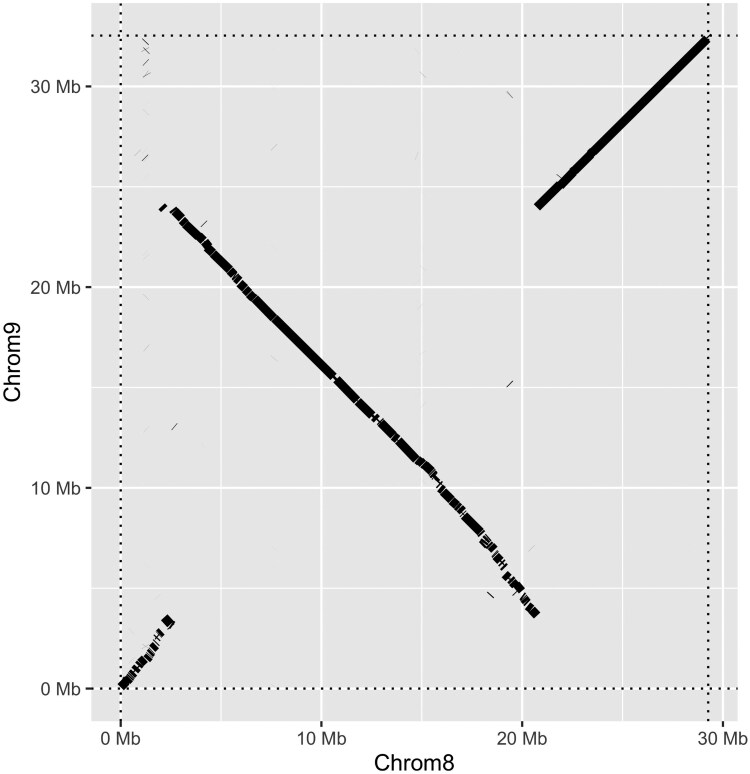
Alignment of chromosome 9 from the *M. merluccius* MmG genome with the homologous chromosome 8 from the MmN genome (*x*-axis values).

The most compelling genomic regions, detected in the sample of 5 males and 5 females through WGS, were analyzed in a large sample to confirm their association with sex. Twenty-five SNPs from these regions were selected for genotyping in 119 individuals (56 males and 63 females) coming from the same population using the MassARRAY technology (Supplementary Table 8). Allelic variants at all the SNPs conformed to the in silico data from WGS, and genotyping was highly consistent, with no missing data across the >3,000 genotypes obtained (Supplementary Table 9). Association with sex was highly significant for most SNPs at C9 according to an XX/XY system, consistent with the WGS data, although the association was not perfect. SNPs 9_13213378 and 9_13224545 showed highly significant differentiation between males and females (*F*_ST_ = 0.223; *P* < 0.001) and a high heterozygote excess associated with males (*F*_IS_ = −0.330; Supplementary Table 10). We also explored LD across 12.0–21.0 Mb of C9 using this set of SNPs and as previously suggested, highly significant LD was detected at very distant positions (>6 Mb) within the region, particularly between 13.2 and 15.1 Mb, where notable genes related to gonad differentiation are located (Fig. 3). Regarding the other 2 regions analyzed at C5 and C12, high genetic differentiation was still maintained in this large sample for 2 SNPs at C5 (5_22355441 and 5_23175612, *F*_ST_ = 0.214; *P* < 0.001), and for the 3 SNPs examined at C12 (12_13762434, 12_18517951, and 12_18533985, *F*_ST_ > 0.170; *P* < 0.001). Furthermore, the putative inversion observed when aligning MmG (female) and MmN (male) genomes (between 4 and 24 Mb; Fig. 4) agrees with the LD detected across the large region at C9 through recombination suppression between X and Y chromosomes.

## Discussion

The chromosome-level genome assembly of *M. merluccius* presented in this study (MmG) represents a significant improvement over the previous version (PRJEB12469; March 2018; scaffold N50: 5.1 kb; total length: 401 Mb; no annotation). Furthermore, the MmG genome demonstrates greater contiguity, completeness, and annotation quality (N50: 32.0 and 715.4 Mb; 26,625 protein-coding genes; 11,083 noncoding transcripts, including 5,683 as lncRNA) compared with the recently released MmN genome (PRJEB77069; September 2024: N50: 28.9 and 647 Mb; 22,124 protein-coding genes). The larger genome size of the MmG is primarily attributed to its higher contiguity at the chromosome ends, while the enhanced annotation likely reflects the integration of RNA-seq data from 5 tissues using pooled samples. Alignment of the MmG and MmN genomes suggests the presence of multiple inversions, not only at chromosome ends, which could reflect technical assembly artifacts, but also at interstitial regions of several chromosomes. Validation of these inversions at the population level will require whole-genome resequencing or PCR assays targeting the inversion breakpoints (Hanlon *et al*. 2022). Inversions, and structural variants in general, make an essential part of genome variation of species that have been related to adaptation, evolution of sex, and speciation, because they represent nonrecombinant genomic islands (Wellenreuther and Bernatchez 2018; Berdan *et al*. 2023).

In this context, the establishment of a reference genome for this commercially valuable fish species presents numerous opportunities to strengthen fisheries management. One key benefit is the development of high-resolution genetic markers, such as SNP panels, which can improve the refinement of the population structure and connectivity and support the recognition of biologically relevant management units that may not correspond to existing administrative stock boundaries (Casas *et al*. 2023). In addition, genomic tools allow for the detection of loci under selection across environmental gradients, offering insights into local adaptation and enabling more targeted, adaptive conservation strategies (Layton *et al*. 2024). A reference genome also facilitates long-term monitoring of genetic diversity and inbreeding, which is especially important in overexploited populations, and helps assess the genetic consequences of practices such as intensive fishing or hatchery releases. Finally, integrating genomic information into eco-genetic models allows researchers and managers to simulate population responses to environmental change and anthropogenic stressors, thereby providing a powerful tool for evidence-based decision-making in fisheries policy and management.

The identification of sex-specific genetic markers, thereby facilitating the implementation of genomic tools for sex determination in fisheries monitoring (Rodríguez-Mendoza and Saborido-Rey 2023), is particularly important in species exhibiting strong sexual dimorphism, such as European hake, where females experience higher fishing mortality due to size-selective fishing pressure and are consequently more vulnerable to adaptive selection and overexploitation. We followed a cost-effective strategy to identify the SD region in *M. merluccius*, which made it possible to suggest an SD system and a putative candidate gene, as successfully demonstrated in other fish species (Martínez *et al*. 2021; De la Herrán *et al*. 2023). A consistent, contiguous, and well-annotated genome, such as the one here presented, is an essential reference for this strategy, ensuring completeness across all chromosomal regions, including centromeres and telomeres. Resequencing a small number of males and females at appropriate depth (30× in this study) would be enough to identify specific genomic regions, not only differentiated between males and females (*F*_ST_ ∼ 0.5) but also involving a significant heterozygote excess (*F*_IS_ ∼ −1) either in males (XX/XY) or females (ZW/ZZ), depending on the SD system. Unlike the pooling strategy frequently used for this purpose (Imarazene *et al*. 2021; Nakamoto *et al*. 2021), which inherently renders false positives, our approach refines candidate regions by simultaneously analyzing both parameters as averages across sliding windows of adjustable size. This strategy necessarily assumes an important genetic component underlying SD, but if this is true, the SD region should be identifiable with a significant reduction of false positives. In fact, we could detect the only genomic region fully compatible with an XX/XY SD system at 14.3 Mb in C9. Furthermore, several other genomic regions showing high differentiation between males and females did not adjust to a typical chromosome SD pattern but instead could represent sexual conflict regions (see below).

Our findings suggest that the main SD region in *M. merluccius* is located on C9, consistent with an XX/XY system, and that *sox3* is the most likely candidate MSD gene. An XX/XY SD system has also been reported in other Gadiformes of the family Gadidae, *Gadus morhua*, *Gadus macrocephalus*, and *Arctogadus glacialis*, but relying on a different MSD gene (*zkY*; Kirubakaran *et al*. 2019). Several lines of evidence support these observations: (1) *F*_ST_ and *F*_IS_ values are above and below the established thresholds, close to the expected 0.5 and −1, respectively; maximum differentiation was observed near *sox3*, where most SNPs were homozygous in females and heterozygous in males; (2) highly significant LD was detected between SNP markers across an extensive region of C9, including the putative MSD gene, in a large sample of males and females; this LD pattern aligns well with the presence of a putative ∼20 Mb inversion encompassing the SD region, potentially suppressing crossing-over between the X and Y chromosomes; (3) several other genes involved in gonad differentiation mapped within the inversion, ensuring their co-transmission as a block linked to the X and Y chromosomes. A duplication of *sox3* has been established, as the MSD gene in mammals (Katsura *et al*. 2018); furthermore, it has also been reported but not related to gene duplication as the MSD gene in several fish species within the genus *Oryzias* (Takehana *et al*. 2014; Myosho *et al*. 2015). Takehana *et al*. (2014) demonstrated that a *cis*-regulatory element located downstream of *sox3* drives increasing expression, triggering testis development via *gsdf*, a member of the transforming growth factor beta family associated with SD in several teleost (Kitano *et al*. 2024). In our study, we did not identify specific allelic variants associated with the X and Y alleles of *sox3*, nor evidence of a gene duplication linked to the Y chromosome. However, the main difference between the X and Y chromosomes was located 25 kb downstream *sox3*, suggesting a potential regulatory element. Further investigation on gene expression of *sox3* in males and females during early stages of gonad differentiation will be required to confirm this hypothesis.

Additional factors are required to fully elucidate the mechanism underlying SD in *M. merluccius*. Validation of SNPs located in the region most strongly associated with sex, using a large sample of 119 males and females, revealed highly significant associations consistent with an XX/XY system, but a perfect genotype–sex correlation was not observed. Disentangling SD in wild populations is complex due to potential interactions between genetic factors and environmental cues across the species’ large distribution area, which can manifest as intraspecific variation in the SD system. Significant variation in the SD systems is often linked to specific environmental factors and has been documented across the geographic ranges of several fish species, such as the Northern pike (*E. lucius*; Johnson *et al*. 2024), flathead gray mullet (*M. cephalus*; Ferraresso *et al*. 2021), Atlantic silverside (*Menidia menidia*; Lagomarsino and Conover 1993), and Argentinian pejerrey (*Odontesthes bonariensis*; Del Fresno *et al*. 2023), among others. Notably, many species where SD systems have been characterized correspond to aquaculture or to model species, usually reared in stable environments. Changes in the environmental context can significantly influence SD systems, as demonstrated in zebrafish, where the original ZW/ZZ system was lost under domesticated conditions (Kossack and Draper 2019). For *M. merluccius*, the influence of environmental factors on a potentially nonfully penetrant genetic system cannot be excluded. Preliminary data suggest differences in the SD system between distant populations across the species’ Atlantic distribution range, namely Norway and Galicia (Martínez *et al*. 2024). These populations exhibit low but significant genetic divergence based on neutral markers (*F*_ST_ = 1.6%; Westgaard *et al*. 2017). Intriguingly, the Northwestern Iberian shelf is the only known region where European hake displays several spawning seasons throughout the year, suggesting potential population subdivision (Serrat *et al*. 2019), which may be critical to fully understand the SD system and its relationship to the broader population structure of European hake in the Atlantic Ocean.

Sexual conflict has been proposed as a key factor driving the evolution of SD systems (Van Doorn and Kirkpatrick 2007). The rapid SD turnover reported in fish represents an excellent model to test this hypothesis. For instance, studies on cichlids from Lake Malawi have identified a novel MSD gene associated with the correct transmission of allelic variants linked to sexually dimorphic coloration patterns favorable to males or females (Roberts *et al*. 2009; Parnell and Streelman 2013). However, beyond color patterns, numerous other traits related to sexual selection or sex-specific reproductive roles could differ between males and females, and manifest as significant genetic differentiation (*F*_ST_) at specific genomic regions. Consequently, associations between genetic markers and sex observed in whole-genome screenings might reflect sexual conflict rather than direct involvement in sex determination, suggesting caution when searching for SD genes, particularly through pooling strategies. In this study, whole-genome screening revealed several regions in different chromosomes, some of them associated with clusters of genes potentially involved in sexual conflict. Given the limited sample size (5 males and 5 females), the risk of false positives was mitigated by validating the observed divergence between sexes in a larger sample set. Among the most divergent regions outside C9, regions at C5 and C12 were prioritized for further validation due to their association with gene clusters related to suggestive traits, such as reproduction and immunity. Several SNPs in these regions maintained the highly significant association observed with sex in a larger sample of 56 males and 63 females (*P* < 0.001). The sex-specific roles in reproduction may explain the associations detected with genes related to olfactory reception and chemosensory communication (Torres *et al*. 2024), functions for which sex dimorphism has been extensively documented, not only in humans (Cherry and Baum 2020) but also in fish species (Kawaguchi *et al*. 2014; Shinohara and Kobayashi 2020; Wang *et al*. 2020). Similarly, sex differences in immune responses, widely studied in humans (Klein and Flanagan 2016; Takahashi *et al*. 2020), have also been reported in fish (Campbell *et al*. 2021). While many of these physiological differences are influenced by sex hormones, the role of selection on alternative genetic variants should not be discarded. These observations, and especially the proposed interpretations, would deserve further work for their confirmation, but they suggest an interesting hypothesis to be tested when performing WGS at the individual level.

### Conclusions

Here, we present a chromosome-level genome assembly of *M. merluccius*, which outperforms previous versions regarding contiguity, completeness, and annotation. This resource represents a reference for population genomics studies aimed at understanding the adaptation of European hake to environmental variation through high-throughput SNP genomic screening and by characterizing the chromosomal inversions here suggested. This highly contiguous genome enabled to screen a sample of males and females by WGS suggesting an XX/XY SD system in this species putatively driven by *sox3*. The nonperfect association in a large population sample suggests that environmental factors or intraspecific variation in the SD system may also be operating, and further studies will be necessary across the whole species range to confirm our observations. Our study also suggests that potential sexual conflicts should be considered when conducting genomic screenings in SD studies. This could provide insights into the high evolutionary turnover of SD in fish.

## Supplementary Material

jkaf127_Supplementary_Data

## Data Availability

All the sequencing data and assembly of *M. merluccius* genome produced in this study have been deposited in the ENA (European Nucleotide Archive); http://www.ebi.ac.uk/ena/browser/view/ under the project accession number PRJEB83726. The genome assembly accession is GCA_964660975.1. SNP genotyping data have been included in Supplementary Table 9. Supplementary materials including Supplementary Tables 2a and 5  Supplementary Figs. 4 and 5 have been deposited in GSA's Figshare and are accessible at https://doi.org/10.25387/g3.28452146. Supplemental material available at G3 online.

## References

[jkaf127-B1] Alioto T, Blanco E, Parra G, Guigó R. 2018. Using geneid to identify genes. Curr Protoc Bioinformatics. 64(1):e56. doi:10.1002/cpbi.56. 30332532

[jkaf127-B2] Altschul SF, Gish W, Miller W, Myers EW, Lipman DJ. 1990. Basic local alignment search tool. J Mol Biol. 215(3):403–410. doi:10.1016/S0022-2836(05)80360-2. 2231712

[jkaf127-B3] Apostologamvrou C, Vlachou M, Theocharis A, Ntavaros C, Klaoudatos D. 2023. Reproductive aspects of European hake, (*Merluccius merluccius*, Linnaeus, 1758) based on histological depiction of both sexes in the Eastern Mediterranean (Aegean Sea). Reg Stud Mar Sci. 68:103281. doi:10.1016/j.rsma.2023.103281.

[jkaf127-B4] Bachtrog D, Mank JE, Peichel CL, Kirkpatrick M, Otto SP, Ashman TL, Hahn MW, Kitano J, Mayrose I, Ming R, et al 2014. Sex determination: why so many ways of doing it? PLoS Biol. 12(7):e1001899. doi:10.1371/journal.pbio.1001899. 24983465 PMC4077654

[jkaf127-B5] Berdan EL, Barton NH, Butlin R, Charlesworth B, Faria R, Fragata I, Gilbert KJ, Jay P, Kapun M, Lotterhos KE, et al 2023. How chromosomal inversions reorient the evolutionary process. J Evol Biol. 36(12):1761–1782. doi 10.1111/jeb.14242. 37942504

[jkaf127-B6] Campbell JH, Dixon B, Whitehouse LM. 2021. The intersection of stress, sex and immunity in fishes. Immunogenetics. 73(1):111–129. doi:10.1007/s00251-020-01194-2. 33426582

[jkaf127-B7] Casas L, Hanel R, Piferrer F, Saborido-Rey F. 2023. Editorial: prospects and challenges for the implementation of HTS genetic methods in fisheries research surveys and stock assessments. Front Mar Sci. 10:1238133. doi:10.3389/fmars.2023.1238133.

[jkaf127-B8] Casey J, Pereiro J. 1995. European Hake (*M. merluccius*) in the North-east Atlantic. In: Alheit J, Pitcher TJ, editors. Hake. Chapman and Hall Fish and Fisheries Series. Vol. 15. Dordrecht: Springer. p. 125–147. doi:10.1007/978-94-011-1300-7_5.

[jkaf127-B9] Cerviño S . 2014. Estimating growth from sex ratio-at-length data in species with sexual size dimorphism. Fish Res. 160:112–119. doi:10.1016/j.fishres.2013.11.010.

[jkaf127-B10] Challis R, Richards E, Rajan J, Cochrane G, Blaxter M. 2020. BlobToolKit—interactive quality assessment of genome assemblies. G3 (Bethesda). 10:1361–1374. doi:10.1534/g3.119.400908. 32071071 PMC7144090

[jkaf127-B11] Chan PP, Lowe TM. 2019. tRNAscan-SE: searching for tRNA genes in genomic sequences. Methods Mol Biol. 1962:1–14. doi:10.1007/978-1-4939-9173-0_1. 31020551 PMC6768409

[jkaf127-B12] Chang CC, Chow CC, Tellier LCAM, Vattikuti S, Purcell SM, Lee JJ. 2015. Second-generation PLINK: rising to the challenge of larger and richer datasets. GigaScience. 4:7. doi:10.1186/s13742-015-0047-8. 25722852 PMC4342193

[jkaf127-B13] Chen S, Zhou Y, Chen Y, Gu J. 2018. Fastp: an ultra-fast all-in-one FASTQ preprocessor. Bioinformatics. 34:i884–i890. doi:10.1093/bioinformatics/bty560. 30423086 PMC6129281

[jkaf127-B14] Cherry JA, Baum MJ. 2020. Sex differences in main olfactory system pathways involved in psychosexual function. Genes Brain Behav. 19:e12618. doi:10.1111/gbb.12618. 31634411

[jkaf127-B15] Cioffi MB, Yano CF, Sember A, Bertollo LAC. 2017. Chromosomal evolution in lower vertebrates: sex chromosomes in neotropical fishes. Genes (Basel). 8:258. doi:10.3390/genes8100258. 28981468 PMC5664108

[jkaf127-B16] Cui X, Lu Z, Wang S, Wang J, Gao X. 2016. CMsearch: simultaneous exploration of protein sequence space and structure space improves not only protein homology detection but also protein structure prediction. Bioinformatics. 32:i332–i340. doi:10.1093/bioinformatics/btw271. 27307635 PMC4908355

[jkaf127-B17] De la Herrán R, Hermida M, Rubiolo JA, Gómez-Garrido J, Cruz F, Robles F, Navajas-Pérez R, Blanco A, Villamayor PR, Torres D, et al 2023. A chromosome-level genome assembly enables the identification of the follicule stimulating hormone receptor as the master sex-determining gene in the flatfish *Solea senegalensis*. Mol Ecol Resour. 23:886–904. doi:10.1111/1755-0998.13750. 36587276

[jkaf127-B18] Del Fresno PS, Garcia de Souza JR, Colautti DC, Yamamoto Y, Yokota M, Strüssmann CA, Miranda LA. 2023. Sex reversal of pejerrey (*Odontesthes bonariensis*), a species with temperature-dependent sex determination, in a seminatural environment. J Fish Biol. 102:75–82. doi:10.1111/jfb.15241. 36217918

[jkaf127-B19] Dobin A, Davis CA, Schlesinger F, Drenkow J, Zaleski C, Jha S, Batut P, Chaisson M, Gingeras TR. 2013. STAR: ultrafast universal RNA-seq aligner. Bioinformatics. 29:15–21. doi:10.1093/bioinformatics/bts635. 23104886 PMC3530905

[jkaf127-B20] Domínguez-Petit R, Saborido-Rey F. 2010. New bioenergetic perspective of European hake (*Merluccius merluccius* L.) reproductive ecology. Fisheries Res. 104:83–88. doi:10.1016/j.fishres.2009.09.002.

[jkaf127-B21] El Bouzidi C, Abid N, Awadh H, Bakkli M, Zerrouk MH. 2022. Growth and mortality of the European hake *Merluccius merluccius* (Linnaeus, 1758) from the North of Moroccan Atlantic coasts. Egypt J Aquat Res. 48:233–239. doi:10.1016/j.ejar.2021.12.003.

[jkaf127-B22] FAO . 2020. The state of the Mediterranean and Black Sea fisheries 2020. Rome, Italy: General Fisheries Commission for the Mediterranean. doi:10.4060/cb2429en.

[jkaf127-B23] Ferraresso S, Bargelloni L, Babbucci M, Cannas R, Follesa MC, Carugati L, Melis R, Cau A, Koutrakis M, Sapounidis A, et al 2021. Fshr: a fish sex-determining locus shows variable incomplete penetrance across flathead grey mullet populations. iScience. 24:101886. doi:10.1016/j.isci.2020.101886. 33354664 PMC7744951

[jkaf127-B24] Gao Y, Wang F, Wang R, Kutschera E, Xu Y, Xie S, Wang Y, Kadash-Edmondson KE, Lin L, Xing Y. 2023. ESPRESSO: robust discovery and quantification of transcript isoforms from error-prone long-read RNA-seq data. Sci Adv. 9:eabq5072. doi:10.1126/sciadv.abq5072. 36662851 PMC9858503

[jkaf127-B25] García-Souto D, Troncoso T, Pérez M, Pasantes JJ. 2015. Molecular cytogenetic analysis of the European hake *Merluccius merluccius* (Merlucciidae, Gadiformes): U1 and U2 snRNA gene clusters map to the same location. PLoS One. 10:e0146150. doi:10.1371/journal.pone.0146150. 26716701 PMC4696792

[jkaf127-B26] Gomez-Garrido J . 2024. CLAWS (CNAG's long-read assembly workflow in Snakemake). WorkflowHub. doi:10.48546/WORKFLOWHUB.WORKFLOW.567.2.

[jkaf127-B27] Guan D, McCarthy SA, Wood J, Howe K, Wang Y, Durbin R. 2020. Identifying and removing haplotypic duplication in primary genome assemblies. Bioinformatics. 36:2896–2898. doi:10.1093/bioinformatics/btaa025. 31971576 PMC7203741

[jkaf127-B28] Guiguen Y, Fostier A, Herpin A. 2019. Sex determination and differentiation in fish: genetic, genomic, and endocrine aspects. In: Wang H-P, Piferrer F, Chen S-L, Shen Z-G, editors. Sex Control in Aquaculture. Vol. 1. New York: John Wiley and Sons Ltd. p. 35–63.

[jkaf127-B29] Haas BJ, Salzberg SL, Zhu W, Pertea M, Allen JE, Orvis J, White O, Buell CR, Wortman JR. 2008. Automated eukaryotic gene structure annotation using EVidenceModeler and the program to assemble spliced alignments. Genome Biol. 9:R7. doi:10.1186/gb-2008-9-1-r7. 18190707 PMC2395244

[jkaf127-B30] Hanlon VCT, Lansdorp PM, Guryev V. 2022. A survey of current methods to detect and genotype inversions. Hum Mutat. 43:1576–1589. doi:10.1002/humu.24458. 36047337

[jkaf127-B31] Imarazene B, Du K, Beille S, Jouanno E, Feron R, Pan Q, Torres-Paz J, Lopez-Roques C, Castinel A, Gil L, et al 2021. A supernumerary “B-sex” chromosome drives male sex determination in the Pachón cavefish, *Astyanax mexicanus*. Curr Biol. 31:4800–4809.e9. doi:10.1016/j.cub.2021.08.030. 34496222 PMC8578452

[jkaf127-B32] Izquierdo F, Paradinas I, Cerviño S, Conesa D, Alonso-Fernández A, Velasco F, Preciado I, Punzón A, Saborido-Rey F, Pennino MG. 2021. Spatio-temporal assessment of the European hake (*Merluccius merluccius*) recruits in the Northern Iberian Peninsula. Front Mar Sci. 8:614675. doi:10.3389/fmars.2021.614675.

[jkaf127-B33] Johnson HA, Rondeau EB, Sutherland BJG, Minkley DR, Leong JS, Whitehead J, Despins CA, Gowen BE, Collyard BJ, Whipps CM, et al 2024. Loss of genetic variation and ancestral sex determination system in North American northern pike characterized by whole-genome resequencing. G3 (Bethesda) Genes, Genomes, Genetics. 14:jkae183. doi:10.1093/g3journal/jkae183. 39115373 PMC11457062

[jkaf127-B34] Katsura Y, Kondo HX, Ryan J, Harley V, Satta Y. 2018. The evolutionary process of mammalian sex determination genes focusing on marsupial SRYs. BMC Evol Biol. 18:3. doi:10.1186/s12862-018-1119-z. 29338681 PMC5771129

[jkaf127-B35] Kawaguchi Y, Nagaoka A, Kitami A, Mitsuhashi T, Hayakawa Y, Kobayashi M. 2014. Gender-typical olfactory regulation of sexual behavior in goldfish. Front Neurosci. 8:91. doi:10.3389/fnins.2014.00091. 24817840 PMC4012221

[jkaf127-B36] Kirubakaran TG, Andersen O, De Rosa MC, Andersstuen T, Hallan K, Kent MP, Lien S. 2019. Characterization of a male specifc region containing a candidate sex determining gene in Atlantic cod. Sci Rep. 9:116. doi:10.1038/s41598-018-36748-8. 30644412 PMC6333804

[jkaf127-B37] Kitano J, Ansai S, Takehana Y, Yamamoto Y. 2024. Diversity and convergence of sex-determination mechanisms in Teleost fish. Annu Rev Anim Biosci. 12:233–259. doi:10.1146/annurev-animal-021122-113935. 37863090

[jkaf127-B38] Klein S, Flanagan K. 2016. Sex differences in immune responses. Nat Rev Immunol. 16:626–638. doi:10.1038/nri.2016.90. 27546235

[jkaf127-B39] Kossack ME, Draper BW. 2019. Genetic regulation of sex determination and maintenance in zebrafish (*Danio rerio*). Curr Top Dev Biol. 134:119–149. doi:10.1016/bs.ctdb.2019.02.004. 30999973 PMC6894417

[jkaf127-B40] Kutsyn DN, Tamoykin IY, Vdodovich IV, Klimova TN, Donchik PI. 2024. Finding of the European hake *Merluccius merluccius* (Merlucciidae) off the Black Sea shore of Crimea. J Ichthyol. 64:80–89. doi:10.1134/S003294522401003X.

[jkaf127-B41] Lagomarsino IV, Conover DO. 1993. Variation in environmental and genotypic sex-determining mechanisms across a latitudinal gradient in the fish, *Menidia menidia*. Evolution. 47:487–494. doi:10.1111/j.1558-5646.1993.tb02108.x. 28568738

[jkaf127-B42] Layton KKS, Brieuc MSO, Castilho R, Diaz-Arce N, Estévez-Barcia D, Fonseca VG, Fuentes-Pardo AP, Jeffery NW, Jiménez-Mena B, Junge C, et al 2024. Predicting the future of our oceans—evaluating genomic forecasting approaches in marine species. Glob Chang Biol. 30:e17236. doi:10.1111/GCB.17236. 38519845

[jkaf127-B43] Li H . 2018. Minimap2: pairwise alignment for nucleotide sequences. Bioinformatics. 34:3094–3100. doi:10.1093/bioinformatics/bty191. 29750242 PMC6137996

[jkaf127-B44] Li H . 2023. Protein-to-genome alignment with miniprot. Bioinformatics. 39:btad014. doi:10.1093/bioinformatics/btad014. 36648328 PMC9869432

[jkaf127-B45] Li H, Durbin R. 2009. Fast and accurate short read alignment with Burrows–Wheeler transform. Bioinformatics. 25:1754–1760. doi:10.1093/bioinformatics/btp324. 19451168 PMC2705234

[jkaf127-B46] Li H, Handsaker B, Wysoker A, Fennell T, Ruan J, Homer N, Marth G, Abecasis G, Durbin R, 1000 Genome Project Data Processing Subgroup. 2009. The sequence alignment/map format and SAMtools. Bioinformatics. 25:2078–2079. doi:10.1093/bioinformatics/btp352. 19505943 PMC2723002

[jkaf127-B47] Lomsadze A, Burns PD, Borodovsky M. 2014. Integration of mapped RNA-seq reads into automatic training of eukaryotic gene finding algorithm. Nucleic Acids Res. 42:e119. doi:10.1093/nar/gku557. 24990371 PMC4150757

[jkaf127-B48] Manni M, Berkeley MR, Seppey M, Simão FA, Zdobnov EM. 2021. BUSCO update: novel and streamlined workflows along with broader and deeper phylogenetic coverage for scoring of eukaryotic, prokaryotic, and viral genomes. Mol Biol Evol. 38:4647–4654. doi:10.1093/molbev/msab199. 34320186 PMC8476166

[jkaf127-B49] Martínez P, Petit N, Blanco A, Carballeda M, Gómez-Garrido J, Cruz F, Alioto T, Saborido F, Casas L. 2024. Sex Determination in Fish: New Insights from the European Hake Merluccius merluccius Chromosome-level Genome Assembly (Oral Communication). Copenhagen (Denmark): AQUA2024-World Aquaculture Society.

[jkaf127-B50] Martínez P, Robledo D, Taboada X, Blanco A, Moser M, Maroso F, Hermida M, Gómez-Tato A, Álvarez-Blázquez B, Cabaleiro S, et al 2021. A genome-wide association study, supported by a new chromosome-level genome assembly, suggests sox2 as a main driver of the undifferentiated ZZ/ZW sex determination of turbot (*Scophthalmus maximus*). Genomics. 113:1705–1718. doi:10.1016/j.ygeno.2021.04.007. 33838278

[jkaf127-B51] Martínez P, Viñas AM, Sánchez L, Díaz N, Ribas L, Piferrer F. 2014. Genetic architecture of sex determination in fish: applications to sex ratio control in aquaculture. Front Genet. 5:340. doi:10.3389/fgene.2014.00340. 25324858 PMC4179683

[jkaf127-B52] Morales-Nin B, Pérez-Mayol S, MacKenzie K, Catalán IA, Palmer M, Kersaudy T, Mahé K. 2022. European hake (*Merluccius merluccius*) stock structure in the Mediterranean as assessed by otolith shape and microchemistry. Fish Res. 254:106419. doi:10.1016/j.fishres.2022.106419.

[jkaf127-B53] Murua H . 2010. The biology and fisheries of European hake, *Merluccius merluccius*, in the North-East Atlantic. Adv Mar Biol. 58:97–154. doi:10.1016/B978-0-12-381015-1.00002-2. 20959157

[jkaf127-B54] Myosho T, Takehana Y, Hamaguchi S, Sakaizumi M. 2015. Turnover of sex chromosomes in celebensis group medaka fishes. G3 (Bethesda). 5:2685–2691. doi:10.1534/g3.115.021543. 26497145 PMC4683641

[jkaf127-B55] Nakamoto M, Uchino T, Koshimizu E, Kuchiishi Y, Sekiguchi R, Wang L, Sudo R, Endo M, Guiguen Y, Schartl M, et al 2021. A Y-linked anti-Müllerian hormone type-II receptor is the sex-determining gene in ayu, *Plecoglossus altivelis*. PLoS Genet. 17:e1009705. doi:10.1371/journal.pgen.1009705. 34437539 PMC8389408

[jkaf127-B56] Nawrocki EP, Eddy SR. 2013. Infernal 1.1: 100-fold faster RNA homology searches. Bioinformatics. 29:2933–2935. doi:10.1093/bioinformatics/btt509. 24008419 PMC3810854

[jkaf127-B57] Niknafs YS, Pandian B, Iyer HK, Chinnaiyan AM, Iyer MK. 2017. TACO produces robust multisample transcriptome assemblies from RNA-seq. Nat Methods. 14:68–70. doi:10.1038/nmeth.4078. 27869815 PMC5199618

[jkaf127-B58] Pan O, Feron R, Jouanno E, Darras H, Herpin A, Koop B, Rondeau E, Goetz FW, Larson WA, Bernatchez L, et al 2021. The rise and fall of the ancient northern pike master sex-determining gene. eLife. 10:e62858. doi:10.7554/eLife.62858. 33506762 PMC7870143

[jkaf127-B59] Parnell N, Streelman J. 2013. Genetic interactions controlling sex and color establish the potential for sexual conflict in Lake Malawi cichlid fishes. Heredity (Edinb). 110:239–246. doi:10.1038/hdy.2012.73. 23092997 PMC3668650

[jkaf127-B60] Pertea M, Pertea GM, Antonescu CM, Chang TC, Mendell JT, Salzberg SL. 2015. StringTie enables improved reconstruction of a transcriptome from RNA-seq reads. Nat Biotechnol. 33:290–295. doi:10.1038/nbt.3122. 25690850 PMC4643835

[jkaf127-B61] Piñeiro C, Saínza M. 2003. Age estimation, growth and maturity of the European hake (*Merluccius merluccius* (Linnaeus, 1758)) from Iberian Atlantic waters. ICES J Mar Sci. 60:1086–1102. doi:10.1016/S1054-3139(03)00086-9.

[jkaf127-B62] Ramos L, Antunes A. 2022. Decoding sex: elucidating sex determination and how high-quality genome assemblies are untangling the evolutionary dynamics of sex chromosomes. Genomics. 114:110277. doi:10.1016/j.ygeno.2022.110277. 35104609

[jkaf127-B63] Ranallo-Benavidez TK, Jaron KS, Schatz MC. 2020. GenomeScope 2.0 and Smudgeplot for reference-free profiling of polyploid genomes. Nat Commun. 11:1432. doi:10.1038/s41467-020-14998-3. 32188846 PMC7080791

[jkaf127-B64] Raymond M, Rousset F. 1995. GENEPOP (version 1.2): population genetics software for exact tests and ecumenicism. J Hered. 86:248–249. doi:10.1093/oxfordjournals.jhered.a111573.

[jkaf127-B65] Roberts RB, Ser JR, Kocher TD. 2009. Sexual conflict resolved by invasion of a novel sex determiner in Lake Malawi cichlid fishes. Science. 26:998–1001. doi:10.1126/science.1174705. PMC3174268 19797625

[jkaf127-B66] Rodríguez-Mendoza R, Saborido-Rey F. 2023. The potential use of genomic methods in bottom trawl surveys to improve stock assessments in Europe. Front Mar Sci. 10:1095171. doi:10.3389/fmars.2023.1095171.

[jkaf127-B67] Serrat A, Saborido-Rey F, Garcia-Fernandez C, Muñoz M, Lloret J, Thorsen A, Kjesbu OS. 2019. New insights in oocyte dynamics shed light on the complexities associated with fish reproductive strategies. Sci Rep. 9:18411. doi:10.1038/s41598-019-54672-3. 31804526 PMC6895218

[jkaf127-B68] Shinohara Y, Kobayashi M. 2020. Sexual bipotentiality of the olfactory pathway for sexual behavior in goldfish. Fish Sci. 86:819–827. doi:10.1007/s12562-020-01454-w.

[jkaf127-B69] Stanke M, Schöffmann O, Morgenstern B, Waack S. 2006. Gene prediction in eukaryotes with a generalized hidden Markov model that uses hints from external sources. BMC Bioinformatics. 7:62. doi:10.1186/1471-2105-7-62. 16469098 PMC1409804

[jkaf127-B70] Takahashi T, Ellingson MK, Wong P, Israelow B, Lucas C, Klein J, Silva J, Mao T, Oh JE, Tokuyama M, et al 2020. Sex differences in immune responses that underlie COVID-19 disease outcomes. Nature. 588:315–320. doi:10.1038/s41586-020-2700-3. 32846427 PMC7725931

[jkaf127-B71] Takehana Y, Matsuda M, Myosho T, Suster ML, Kawakami K, Shin-I T, Kohara Y, Kuroki Y, Toyoda A, Fujiyama A, et al 2014. Co-option of Sox3 as the male-determining factor on the Y chromosome in the fish *Oryzias dancena*. Nat Commun. 5:4157. doi:10.1038/ncomms5157. 24948391

[jkaf127-B72] Toronen P, Holm L. 2022. PANNZER—a practical tool for protein function prediction. Protein Sci. 31:118–128. doi:10.1002/pro.4193. 34562305 PMC8740830

[jkaf127-B73] Torres D, Villamayor PR, Román A, García P, Martínez P, Sánchez-Quinteiro P. 2024. In-depth histological, lectin-histochemical, immunohistochemical and ultrastructural description of the olfactory rosettes and olfactory bulbs of turbot (*Scophthalmus maximus*). Cell Tissue Res. 397:215–239. doi:10.1007/s00441-024-03906-6. 39112611

[jkaf127-B74] Van Doorn GS, Kirkpatrick M. 2007. Turnover of sex chromosomes induced by sexual conflict. Nature. 449:909–912. doi:10.1038/nature06178. 17943130

[jkaf127-B75] Wang J, Tao W, Kocher TD, Wang D. 2024. Sex chromosome turnover and biodiversity in fishes. J Genet Genom. 51:1351–1360. doi:10.1016/j.jgg.2024.08.008. 39233051

[jkaf127-B76] Wang Y, Jiang H, Yang L. 2020. Transcriptome analysis of zebrafish olfactory epithelium reveals sexual differences in odorant detection. Genes (Basel). 11:592. doi:10.3390/genes11060592. 32471067 PMC7349279

[jkaf127-B77] Wellenreuther M, Bernatchez L. 2018. Eco-evolutionary genomics of chromosomal inversions. Trends Ecol Evol. 33:427–440. doi:10.1016/j.tree.2018.04.002. 29731154

[jkaf127-B78] Westgaard JI, Staby A, Godiksen JA, Geffen AJ, Svensson A, Charrier G, Svedäng H, André C. 2017. Large and fine scale population structure in European hake (*Merluccius merluccius*) in the northeast Atlantic. ICES J Mar Sci. 74:1300–1310. doi:10.1093/icesjms/fsw249.

[jkaf127-B79] Wickham H . 2016. ggplot2: Elegant Graphics for Data Analysis. Vienna: Springer-Verlag. doi:10.1007/978-3-319-24277-4.

[jkaf127-B80] Winter D. 2020. pafr: Read, Manipulate and Visualize ‘Pairwise mApping Format’ Data_. R package version 0.0.2. https://CRAN.R-project.org/package=pafr.

[jkaf127-B81] Wright S . 1949. The genetical structure of populations. Ann Eugen. 15:323–354. doi:10.1111/j.1469-1809.1949.tb02451.x. 24540312

[jkaf127-B82] Zhou C, McCarthy SA, Durbin R. 2023. YaHS: yet another Hi–C scaffolding tool. Bioinformatics. 39:btac808. doi:10.1093/bioinformatics/btac808. 36525368 PMC9848053

