# Supplementary material for: Screening a new European hake (Merluccius merluccius) chromosome-level genome assembly suggests an XX/XY sex-determining system driven by the SRY-box transcription factor 3 (sox3)
Source: G3 (Bethesda). 2025 Jun 9;15(8):jkaf127. doi: 10.1093/g3journal/jkaf127 (PMC12341869; doi:10.1093/g3journal/jkaf127)
Supplement: jkaf127_Supplementary_Data [file jkaf127_supplementary_data.zip › Supplemental_Figures_S1-S3_G3-2025-405928.docx]

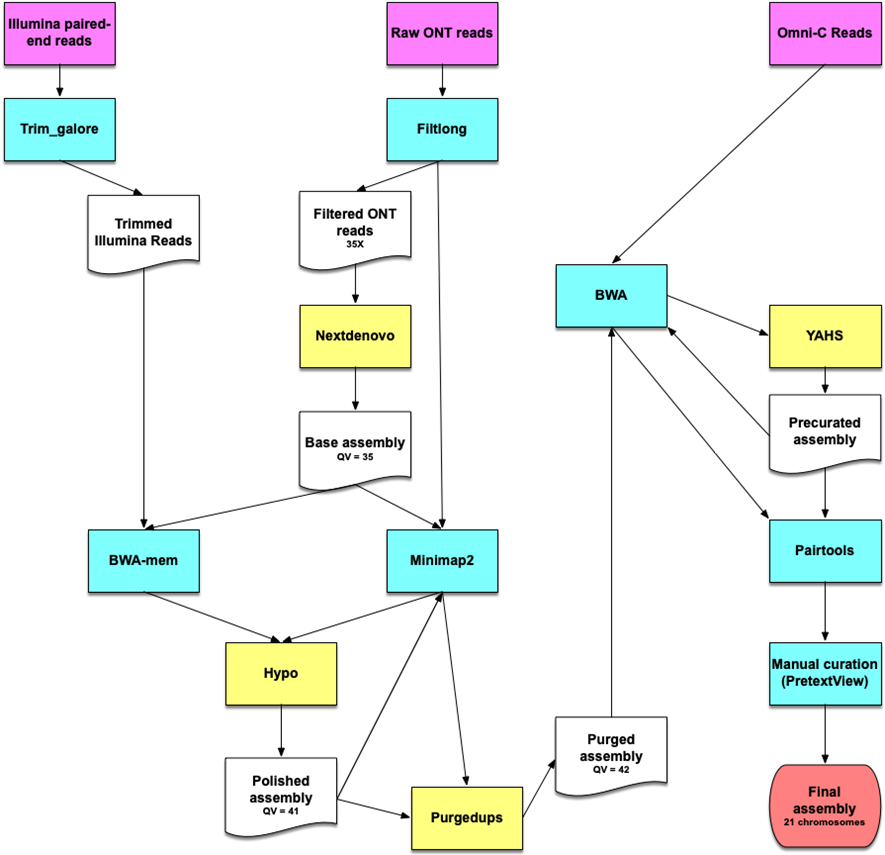


**Figure S1:** Workflow of the *M*. *merluccius* genome assembly

**Figure S2:** Flowchart of the annotation process of the *M*. *merluccius* genome.


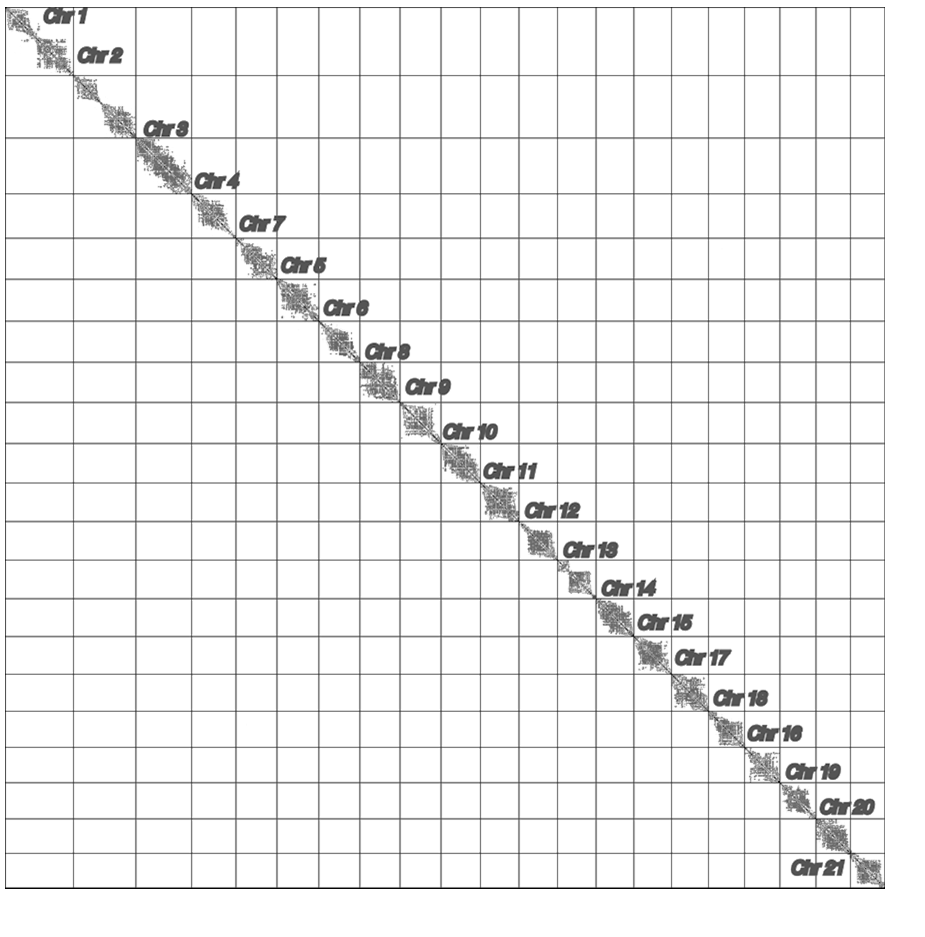


**Figure S3**. Scaffolding of the *M*. *merluccius* genome through chromatin contacts obtained by Omni-C from different tissues. Note the two arms in the metacentric chromosomes 2, 3 and 14 in accordance with the species karyotype (García-, 2015)
