# Supplementary material for: Screening a new European hake (Merluccius merluccius) chromosome-level genome assembly suggests an XX/XY sex-determining system driven by the SRY-box transcription factor 3 (sox3)
Source: G3 (Bethesda). 2025 Jun 9;15(8):jkaf127. doi: 10.1093/g3journal/jkaf127 (PMC12341869; doi:10.1093/g3journal/jkaf127)
Supplement: jkaf127_Supplementary_Data [file jkaf127_supplementary_data.zip › Supplemental_Methods_G3-2025-405928.docx]

**Supplementary Methods**

Long-read Whole Genome Sequencing

4.0 μg of high molecular weight DNA was end-repaired using the NEBNext FFPE DNA Repair Mix (NEB) and the NEBNext UltraII End Repair/dA-Tailing Module NEB. Then, sequencing adaptor ligation, purification by 0.4X AMPure XP Beads and elution in ONT elution buffer (SQK-LSK109) were performed. Sequencing runs were carried out on a GridION Mk1 (ONT) using a Flowcell R9.4.1 FLO-MIN106D (ONT), with sequencing data collected over 110 hours. The quality parameters of the sequencing runs were monitored in real time by the MinKNOW platform version 4.1.2, and base-calling was performed with Guppy version 4.2.3.

Short-read whole genome sequencing

1.0 µg of genomic DNA was sheared on a Covaris™ LE220-Plus (Covaris) and size-selected for fragment sizes of 220-550 bp with AMPure XP beads (Agencourt, Beckman Coulter) and libraries prepared using the KAPA HyperPrep kit (Roche). The genomic DNA fragments were then end-repaired and adenylated. Next, compatible adaptors for Illumina platforms with unique dual indexes and unique molecular identifiers (Integrated DNA Technologies), were ligated. The libraries were quality controlled on an Agilent 2100 Bioanalyser with the DNA 7500 assay (Agilent) for size and quantified using the Kapa Library Quantification Kit for Illumina platforms (Roche).

RNA-Seq

500 ng of total RNA was used for poly-A fraction enrichment with oligo-dT magnetic beads, following the mRNA fragmentation. Strand specificity was achieved during second strand synthesis, which was performed in the presence of dUTP instead of dTTP. The blunt-ended double stranded cDNA was 3´adenylated before ligating Illumina platform-compatible adaptors with unique dual indexes and unique molecular identifiers (Integrated DNA Technologies). The ligation product was enriched using 15 PCR cycles, and the final library was validated on an Agilent 2100 Bioanalyser with the DNA 7500 assay.

Hi-C sequencing

Chromatin from pooled organs was crosslinked with formaldehyde (Sigma Aldrich), digested with DNase I and the DNA was extracted. The DNA ends were repaired, and a biotinylated bridge adapter was ligated, followed by proximity ligation of adapter-containing ends. After reverse crosslinking, the DNA was purified and used to prepare Illumina-compatible paired-end sequencing libraries (omitting the fragmentation step). Biotinylated chimeric molecules were isolated using streptavidin beads prior to PCR enrichment of the library.

MassARRAY

A MassARRAY assay was used for SNP genotyping, utilizing a set of markers located in the candidate regions. The MassARRAY assay consists of two consecutive reactions; the first step involves the PCR amplification of the region containing the target SNP and the second step is a mini-sequencing reaction using an internal primer adjacent to the SNP site, using dideoxy nucleotides. Thus, two flanking PCR primers and one internal primer adjacent to the variable site were designed for genotyping each SNP, taking the flanking sequences from our assembled genome.
